# A Combination of Cytokine-Induced Killer Cells With PD-1 Blockade and ALK Inhibitor Showed Substantial Intrinsic Variability Across Non-Small Cell Lung Cancer Cell Lines

**DOI:** 10.3389/fonc.2022.713476

**Published:** 2022-05-11

**Authors:** Yutao Li, Amit Sharma, Xiaolong Wu, Hans Weiher, Dirk Skowasch, Markus Essler, Ingo G. H. Schmidt-Wolf

**Affiliations:** ^1^ Department of Integrated Oncology, Center for Integrated Oncology (CIO) Bonn, University Hospital Bonn, Bonn, Germany; ^2^ Department of Neurosurgery, University Hospital Bonn, Bonn, Germany; ^3^ Department of Applied Natural Sciences, Bonn-Rhein-Sieg University of Applied Sciences, Rheinbach, Germany; ^4^ Department of Internal Medicine II, Cardiology, Pneumology and Angiology, University Hospital Bonn, Bonn, Germany; ^5^ Department of Nuclear Medicine, University Hospital Bonn, Bonn, Germany

**Keywords:** cytokine-induced killer cells, immune checkpoint inhibition programmed cell death-1, anaplastic lymphoma kinase, immunotherapy, non-small cell lung cancer

## Abstract

**Background:**

Cancer heterogeneity poses a serious challenge concerning the toxicity and adverse effects of therapeutic inhibitors, especially when it comes to combinatorial therapies that involve multiple targeted inhibitors. In particular, in non-small cell lung cancer (NSCLC), a number of studies have reported synergistic effects of drug combinations in the preclinical models, while they were only partially successful in the clinical setup, suggesting those alternative clinical strategies (with genetic background and immune response) should be considered. Herein, we investigated the antitumor effect of cytokine-induced killer (CIK) cells in combination with ALK and PD-1 inhibitors *in vitro* on genetically variable NSCLC cell lines.

**Methods:**

We co-cultured the three genetically different NSCLC cell lines NCI-H2228 (EML4-ALK), A549 (KRAS mutation), and HCC-78 (ROS1 rearrangement) with and without nivolumab (PD-1 inhibitor) and crizotinib (ALK inhibitor). Additionally, we profiled the variability of surface expression multiple immune checkpoints, the concentration of absolute dead cells, intracellular granzyme B on CIK cells using flow cytometry as well as RT-qPCR. ELISA and Western blot were performed to verify the activation of CIK cells.

**Results:**

Our analysis showed that (a) nivolumab significantly weakened PD-1 surface expression on CIK cells without impacting other immune checkpoints or PD-1 mRNA expression, (b) this combination strategy showed an effective response on cell viability, IFN-γ production, and intracellular release of granzyme B in CD3^+^ CD56^+^ CIK cells, but solely in NCI-H2228, (c) the intrinsic expression of Fas ligand (FasL) as a T-cell activation marker in CIK cells was upregulated by this additive effect, and (d) nivolumab induced Foxp3 expression in CD4^+^CD25^+^ subpopulation of CIK cells significantly increased. Taken together, we could show that CIK cells in combination with crizotinib and nivolumab can enhance the anti-tumor immune response through FasL activation, leading to increased IFN-γ and granzyme B, but only in NCI-H2228 cells with EML4-ALK rearrangement. Therefore, we hypothesize that CIK therapy may be a potential alternative in NSCLC patients harboring EML4-ALK rearrangement, in addition, we support the idea that combination therapies offer significant potential when they are optimized on a patient-by-patient basis.

## Introduction

Cancer is a highly dynamic disease where clinical (inter-individual differences), molecular (epigenomics), and yet to be known factors often pose a challenge to find a successful treatment option (1, 2). Although, the traditional therapies (surgery, radiation, and chemotherapy) for cancer are still being used, the maximum therapeutic benefit has been obtained using combination therapies. For instance, Pembrolizumab (anti-PD-1 mAb) in combination with chemotherapy in patients with non-small cell lung cancer (NSCLC) showed better survival compared to those treated only with chemotherapy (3). In a meta-analysis including seven studies (>4000 patients) on pretreated NSCLC, Tartarone et al. showed that among immune checkpoint inhibitors, anti-PD-1 provided greater benefit compared to anti-PD-L1 (4). Similarly, in another independent meta-analysis in which immune checkpoint inhibitors were added to chemotherapy, Petrelli et al. showed that there was a significant overall survival benefit in NSCLC cases with PD-L1 (5). Hence, there is reasonable evidence that modulation of PD-1/PD-L1 associated pathways may have predictive significance for the clinical NSCLC spectrum. Since the combination therapy minimizes the toxic effects on normal cells and induces cytotoxic effects on the cancer cells, it also allows the possibility to further combine more than one inhibitor, especially when considering the mutation/genomic landscape, as shown in a recent clinical trial that combined durvalumab (human IgG1κ monoclonal antibody that blocks PD-L1 binding to PD-1 & CD80) and gefitinib (EGFR tyrosine kinase inhibitor) to treat TKI-naïve patients with EGFR mutation-positive NSCLC (6).

It is also worth mentioning that the effective use of combined cancer therapies remains a challenge, especially when optimization is concerned. This can be evident from a recent clinical trial involving crizotinib (ALK inhibitor) and nivolumab (PD-1 inhibitor) in NSCLC that was discontinued due to the safety concerns (CheckMate 370) (7). However, the clinical trials based on a similar combination strategy such as TKI/ALK inhibitor with ICI are still in progress. Primarily, the concern about short-term gastrointestinal toxicity also emerged in the above-mentioned clinical trial, however, it was resolved with the standard medical care. Notably, the treatment sequence also appeared to be pivotal, as evidenced by the fact that treatment in reverse sequence (osimertinib followed by nivolumab) did not result in higher levels of toxicity compared to treatment with nivolumab followed by osimertinib (8). To mention, the second generation ALK inhibitor ceritinib showed a synergistic effect with PD-1/PD-L1 blockade to provide an improved anti-tumor response along with favorable side effect tolerability *in vivo* NSCLC xenograft model (9) and clinical trial (10). Despite T cells dominated the immune cell composition in NSCLC tumors (11), the function of CD4^+^ and CD8^+^ T lymphocytes was dysregulated with decreasing of IFN-γ production (12). In this particular scenario, the inclusion of alternative adjuvant treatments, for instance, cytokine-induced killer (CIK) cells, may help to reshape the therapeutic paradigm in NSCLC patients. CIK cells are heterogeneous *in vitro* expanded T lymphocytes with a natural killer (NK)/T phenotype generated primarily by *ex vivo* incubation of human peripheral blood mononuclear cells (PBMC) or cord blood mononuclear cells. The transfusion of CIK cells after *ex-vivo* expansion in cancer patients has already been tested in more than 80 reported clinical trials (13). Moreover, recent studies provide functional details on the function and optimization of CIK cells to maximize their functional potential (14–16). Of note, there have been autologous and allogeneic clinical trials showing that CIKs immunotherapy has potential benefits in the safety and efficacy of patients with advanced NSCLC (17–21), given that clinical trials of DC-CIK (dendritic cells cytokine-induced killer cells) in combination with chemotherapy for advanced lung cancer have shown very limited success. To improve the efficiency of such therapies, several paradigms have been discussed, including inhibition of inflammatory mediators released by tumor cells in combination with vaccination to reduce recruitment of tumorigenic immune cells to the tumor microenvironment in pre/postoperative advanced lung cancer (22). Certainly, the DCs loaded with tumor antigens along with CIK cells may have a lower risk compared to CAR‐T cells alone. To mention, the combination of CIK cells with PD-1 blockade before transfusion might improve the efficiency of CIK therapy for NSCLC patients *in vitro* (23). Alternatively, autologous cytokine‐induced killer (CIK) cells enhance the clinical response to PD‐1 blocking antibodies in patients with advanced non‐small-cell lung cancer (24). Recently, the benefits of combining anti-PD-1 antibody with antiangiogenic drugs anlotinib in NSCLC patients have been reported (25). Although there is some previous evidence of ICIs combinations, there has been no report on the combination of CIK with ALK inhibitors and PD-1 inhibitors neither *in vitro* nor *vivo*.

Considering this, we aim to understand whether pretreated-nivolumab CIK cells before transfusion can enhance the antitumor immune response to NSCLC cell lines at different concentrations of crizotinib *in vitro*. To achieve this, we used three NSCLC cell lines: NCI-H2228 (EML4-ALK), A549 (KRAS mutation), and HCC-78 (ROS1 rearrangement) having different genetic alterations and employing multiple techniques (flow cytometry, intracellular staining for granzyme B, cell viability assays, ELISA, RT-PCR, Western blot).

## Material and Methods

### Regents and Antibodies

Anti-PD-1 mAb nivolumab (purity 99.50%) and ALK inhibitor crizotinib (purity ≥98% (HPLC) were purchased from Selleckchem Co., Ltd. (Houston, TX). Crizotinib was dissolved in dimethyl sulfoxide (DMSO). DMSO was used as a control for crizotinib and 20 μg/mL IgG4 isotype (Biolegend, San Diego, CA)as a control for nivolumab. Concerning antibodies (Abs): The fluorochrome-conjugated FITC anti-human CD3 antibody (Clone OKT3), brilliant violet 421 anti-human CD8 antibody (RPA-T8), APC anti-human CD4 antibody (Clone OKT4), PE-CD56 (Clone 5.1 H11), APC anti-human PD1 (Clone NAT105), PE anti-human PD-L1(Clone 29E-2A3), APC anti-human CTLA-4 antibody (Clone L3D10), PE anti-human GITR antibody (Clone 621), brilliant violet 421 anti-human CD134 (OX40) antibody (Clone Ber-ACT35), and FITC anti-human/mouse granzyme B antibody (GB11) were purchased from Biolegend (San Diego, CA). For APC-anti-human PD-1 detection, its isotype control is mouse IgG1k, which is not compatible to nivolumab as an IgG4 antibody. Anti-Human Foxp3 Staining Set FITC kit (eBioscience, San Diego, CA) was used to identify Foxp3 CD4^+^CD25^+^/CD4^+^CD25^-^ cells according to the manufacturer’s instructions. For Western blot: Anti-FasL (Biolegend, San Diego, CA) and beta Tubulin Loading Control Monoclonal Antibody (BT7R) (Thermo Fisher Scientific, San Diego, CA) were used.

### Cell Culture

CIK cells were generated, as previously described (26, 27). PBMCs required for the experiments were isolated from the blood of healthy donors registered at the blood bank of University Hospital Bonn. Three epithelial lung cancer cell lines: A549 cells (KRAS mutation), HCC-78 (ROS1 rearrangement, SLC34A2-ROS1), and NCI-H2228 (EML4-ALK variant 3) were primarily used in this study. All cell lines were mycoplasma negative and cultured in RPMI medium supplemented with 10% heat-inactivated FBS (Sigma-Aldrich GmbH, Munich, Germany) and 1% penicillin/streptomycin (P/S) (Gibco, Germany) at 37°C (5% CO_2_). As mentioned above, to avoid the excessive toxicity of simultaneous treatment of nivolumab and crizotinib, CIK cells were pre-treated with 20 μg/mL anti-PD-1 mAb nivolumab or IgG4 isotype control for 24 h and then co-cultured with the tumor cells along with different concentrations of ALK inhibitor crizotinib.

### Cell Viability Assessment by CCK-8 Assay and Cell Death Analysis byFlow Cytometry

CCK-8 cell viability assay was performed, as described by the manufacturer (Dojindo Laboratories, Kumamoto, Japan). NSCLC cells were seeded into 96-well plates (1x10^4^ cells/well) and treated with various concentrations of crizotinib or DMSO for 24 h in the presence or absence of nivolumab. Similarly, the flow cytometry-based cytotoxicity was performed, as described protocol. Briefly, the target cells were labeled with CFSE (1 x 10^6^ cells in 1 ml PBS with 0.5 uM CFSE, 20 min, 37˚C in the dark) and washed twice with warm culture medium. CFSE-labeled 5 x 10^4^ tumor cells were incubated at various concentrations of crizotinib for 24 h with CIK cells pre-incubated with 20 μg/mL nivolumab or IgG 4 isotype (24 h) to perform redirected cytolysis assay at an E/T ratio of 10:1. Following 24 h of culturing, the cells were stained with Hoechst 33258 (Cayman Chemical, Hamburg, Germany) and were quantified using BD FACS Canto II. The absolute number of 3000 beads (Biolegend, San Diego, CA) was acquired by a BD Canto II cytometer. Then the absolute numbers of cells per uL were analyzed by FlowJo V10 software (Tree Star, Ashland, Oregon). The absolute number of cells were calculated according to Precision Count protocol provided by Biolegend Company as follows: 


$$Absolute\;Cell\;Count\;(Cells/uL)=(Cell\;Count)/(Precision\;Count\;Beads^{TM})x\;Precision\;Count\;Beads^{TM}\;Concentration$$

### Intracellular Staining for Granzyme B by Flow Cytometry

A fixable Viability Zombie Aqua™ Dye (Biolegend, San Diego, CA) was used to exclude dead cells from the analysis. Mainly, after staining the surface receptors with PE anti-human CD3 antibody and APC anti-human CD56, the cells were fixed with 100 μL fix solution (eBioscience, San Diego, CA) for 30 min at room temperature (in the dark). Cells were then washed and resuspended in 100 μL 1x permeabilization buffer and stained with FITC anti-human granzyme B antibody for 30 min at room temperature. Subsequently, the cells were washed twice with 2 mL 1x permeabilization buffer, resuspended in DPBS, and recorded with a BD Canto II cytometer.

### Enzyme-Linked Immunosorbent Assay (ELISA)

The ELISA assay was performed using the standard protocol. Briefly, 1 x 10^6^ CIK cells incubated with 20 μg/mL nivolumab or IgG4 isotype control for 24 h. After that, CIK cells were co-cultured with 5 x 10^4^ tumor cells in the presence of various concentrations of crizotinib for 24 h. Thereafter, the cell-free supernatant was collected to perform sandwich ELISA assay (IFN Gamma Kit, Invitrogen, Camarillo, CA), according to the manufacturer’s instructions.

### Reverse Transcription and Quantifying PCR (RT-qPCR)

CIK cells were incubated with 20 ug/mL nivolumab or IgG4 isotype control for 48 h. Afterward, CIK cells were washed twice with cold DPBS. RNA was extracted using RNeasy^®^ Plus Mini Kit (QIAGEN, Hilden, Germany) and cDNA synthesis was performed by SuperScript™ III First-Strand Synthesis Super Mix Kit (Invitrogen, CA), according to the manufacturer’s instructions. Quantitative real-time PCR analysis was performed using QuantStadio 6 Flex Sequence Detection System (384-well, Applied Biosystems, CA) using SYBR^®^ Select Master Mix (Applied Biosystems, CA). For q-PCR, the following primers were used to amplify PD-1 [as described (28)]: forward primer 5#-CAGGGTGACAGAGAAGGG-3#, reverse primer 5#-CCTGGCTCCTATTGTCCCTC-3#, β-actin: 5#-ACCGCGAGAAGATGACCCAGA3# (forward) and 5#-GGATAGCACAGCCTGGATAGCAA3# (reverse) (obtained by Eurofins Genomics Germany GmbH, Ebersberg, Germany). The relative expression of PD-1 was normalized to β-actin expression by ΔΔCt method. To determine C - reactive protein (CRP) transcription level, the following primers were used to amplify (as described (29)): forward primer 5´- CAGACAGACATGTCGAGGAAGG-3#, reverse primer 5´- AGGCTTTGAGAGGCTTCGTT-3´, HPRT: 5´-TCAGGCAGTATAATCCAAAGATGGT-3´, (forward) and 5´-AGTCTGGCTTATATCCAACACTTCG-3´ (reverse) (obtained by Eurofins Genomics Germany GmbH, Ebersberg, Germany). The relative expression of c-reaction protein (CRP) was normalized to HPRT expression by the ΔΔCt method.

### Western Blot Assay

CIK cells incubated with 20 ug/mL nivolumab and various concentrations of crizotinib for 48 h were washed twice with cold DPBS. Cell pellets were lysed using NuPAGE LDS buffer, lysates were separated on 4-12% Tris-glycine gels and transferred to PVDF membranes. Anti-FasL antibody (Biolegend, San Diego, CA) were used for primary antibody. Beta Tubulin Loading Control Monoclonal Antibody (BT7R) (Thermo Fisher Scientific, Inc. San Diego, CA) was used as a loading control.

### LDH Assay for the Assessment Hepatoxicity

The cytotoxicity of a combined nivolumab and crizotinib simultaneously in the absence or presence of CIK cells on hepatocyte-like cell line CCL-13, which exhibits the liver function of primary human hepatocytes, was detected by CyQUANT™ LDH Cytotoxicity Assay Kit (Invitrogen, Inc. San Diego, CA.). Briefly, we co-cultured CIK with 1x10^4^ CCL-13 cell line at an E:T ratio 1:1 in the presence of crizotinib/nivolumab triplicate in 96-well flat plates for 16 h. We calculated the percentage of hepatoxicity with the following formula:


$$\%\;hepatoxicity=\frac{experimental\;LDH\;activity\;-\;spontaneous\;CCL\;-\;13\;LDH\;activity-spontaneous\;CIK\;LDH\;activity}{Maximum\;LDH\;activity\;-\;spontenous\;CCL\;-13\;LDH\;activity}$$

### Statistical Analysis

All data were presented as the mean ± SD from at least three independent experiments. FACS data sets were analyzed using FlowJo V10.6 software (FlowJo, LLC, Ashland, Oregon). The statistical analysis was performed using SPSS Statistics 23. The data groups were compared using one-way analysis of variance with Turkey *post hoc* test and Student’s t-test. P-values < 0.05 were considered significant differences and are marked: * < 0.05; ** < 0.01; *** < 0.001.

## Results

### Elevated PD-1/PD-L1 Expression in CIK Subsets After 14 Days of *In Vitro* Expansion

**Figure 1:**
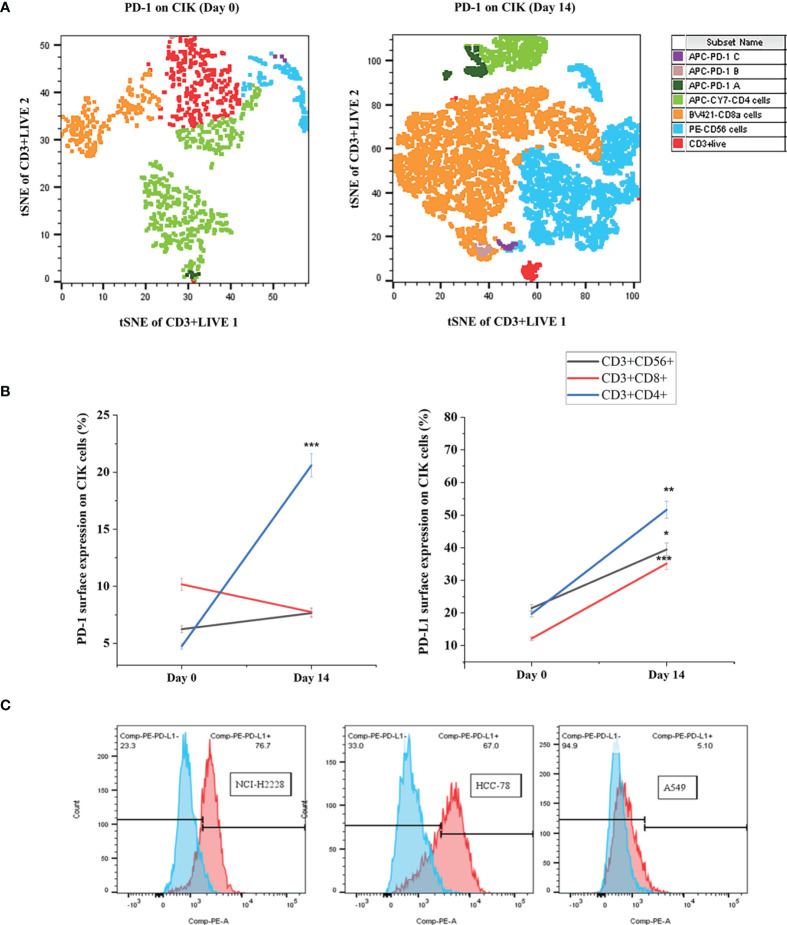
PD-1^+^/PD-L1^+^ surface expression on Day 0 and Day 14 of CIK cells. The differential expression of PD-1/PD-L1 phenotypic subsets of CIK cells over *in vitro* culture is shown by flow cytometric analysis. PBMC were isolated from healthy donors and cultured in the presence of IFN- γ on Day 0, anti-human CD3 antibody, IL-1 β and IL-2 on Day 1. After 14 days of expansion with IL-2 induction, CIK cells were investigated by flow cytometric method by staining with FITC-CD3, PE-CD56, APC-Cy7-CD4, Bv421-CD8, and APC-PD-1. Dead CIK cells were gated excluding by 7-AAD. **(A)** The percentage of PD-1 on Day 0 and Day 14 CIK cells represented by tSNE plots using Flowjo V10 software. APC-PD-1 A represented APC-PD-1^+^ APC-CY7-CD4^+^ cells; APC-PD-1 B represented APC-PD-1^+^BV421-CD8a^+^ cells; APC-PD-1 C represented APC-PD-1^+^PE-CD56^+^ cells. **(B)** Summary data of the frequency of PD-1^+^/PD-L1^+^ CIK subsets in healthy subjects. **(C)** Variation of PD-L1 surface expression on the 3 NSCLC cell lines, measured by flow cytometry. All data are shown as the mean ± SD. * representative of three independent experiments. *P < 0.05, **P < 0.01, ***P < 0.001 vs. Day 0 CIK cells calculated by Student’s t-test. CIK cells derived from three healthy donors.

We have previously shown that CIK cells are heterogeneous and are composed of CD3^+^ CD8^+^, CD3^+^ CD4^+^ and CD3^+^ CD56^+^ specifically on Day 14 (30). Therefore, we first determined the phenotypes of PD-1 and PD-L1 CIK cells primarily on Day 0 and Day 14 for these three CIK subsets. The analysis showed that the percentage of PD-1^+^ CD3^+^ CD4^+^ CIK cells increased significantly after 14 days of expansion compared to Day 0 (**Figures 1A, B**, 20.6 ± 2.0 vs. 4.7 ± 1.0%, P < 0.001). However, no significant difference in the CD3^+^ CD56^+^ and CD3^+^ CD8^+^ CIK cell subsets was observed (6.2 ± 0.7 vs. 7.7 ± 0.7%, P= 0.177; 10.2 ± 2.1 vs. 7.7 ± 0.8%, P= 0.630, respectively). Similarly, the proportion of PD-L1^+^ CIK cells was also increased among the CD3^+^ CD56^+^, CD3^+^ CD8^+^ and CD3^+^ CD4^+^ subsets of CIK cells on Day 14 compared to Day 0 (**Figure 1B**, 39. 5 ± 4.6% vs. 21.4 ± 5.9%, P= 0.025; 35.2 ± 4.5% vs. 12.1 ± 3.3%, P < 0.001; 51.6 ± 8.1% vs. 19.7 ± 4.2%, P= 0.008). To mention, we confirmed that all cell lines expressed PD-L1, but at different levels (**Figure 1C**, NCI-H2228 > HCC-78 > A549, 69.2 ± 8.0% on NCI-H2228, 61.8 ± 4.6% on HCC-78 and 6.0 ± 2.0% on A549).

### Nivolumab Harbors the Potential to Block Surface Expression of PD-1 on CIK Cells

**Figure 2:**
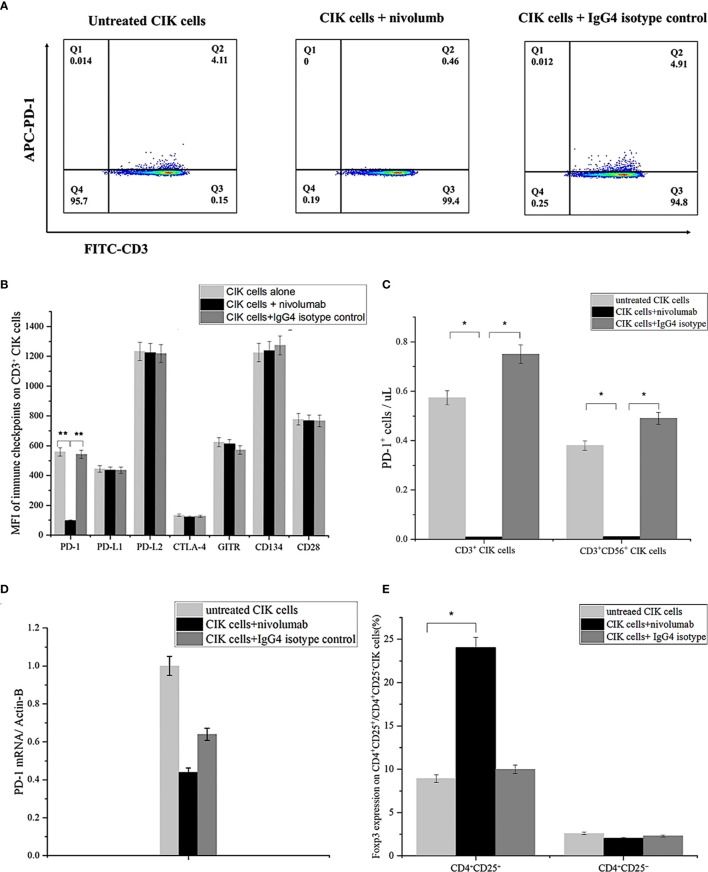
PD-1 expression on CIK cells after treatment with nivolumab for 48 (h) **(A)** PD-1 surface expression on CIK cells in the presence of nivolumab 20 μg/mL for 48 h compared to untreated CIK cells or IgG4 isotype control 20 μg/mL measured by flow cytometry. Dead CIK cells were gated and excluded by 7-AAD. Numbers represent the percentage of the gated population. **(B)** The mean fluorescent intensity (MFI) level of PD-1, PD/L1, PD-L2, CTLA-4, GITR, and CD134 (OX40) on the CD3^+^ CIK cells was observed after 48 h of treatment with nivolumab by flow cytometry. **(C)** Numbers represent the percentage of the gated population. Quantification of the absolute PD-1^+^ CIK cells by count beads. **(D)** Analysis of q-RCR mRNA expression in CIK cells after incubation with nivolumab for 48 (h) PD-1 expression is normalized over β-actin expression. **(E)** Foxp3 expression on CD4^+^ CD25^+^/CD4^+^CD25^-^ CIK cells after 48 h nivolumab treatment. Data from three independent experiments were shown as mean ± SD. Statistical analysis was performed using one‐way ANOVA followed by the Tukey–Kramer *post hoc* test. *p < 0.05, **p < 0.01 vs. untreated CIK cells or CIK cells treated with IgG4 isotype control. CIK cells derived from three healthy donors.

Next, we examined the association between CIK cell activation and PD-1 surface expression in the presence of nivolumab and found that PD-1 expression on the surface of CD3^+^ CIK cells decreased significantly from 6.7 ± 2.7% to 0.4 ± 0.04% after 48 h of treatment with nivolumab compared to the IgG4 isotype control (**Figure 2A**). Also, the assessment of the mean fluorescence intensity (MFI) of surface expression of potential immune-associated markers, including PD-L1, PD-L2, CD28, CTLA-4, GITR, and CD134 on CD3^+^ CIK cells, showed no alterations (**Figure 2B**). When counting the absolute number of PD-1^+^ CIK cells, there was also a notable decrease in both PD-1^+^ CD3^+^ CIK cells and/or PD-1^+^ CD3^+^ CD56^+^ CIK cells after the nivolumab treatment (**Figure 2C**, 0.01 ± 0.01 vs. 0.57 ± 0.18 cells/μL, P= 0.045; 0.01 ± 0.01 vs. 0.38 ± 0.13 cells/μL, P= 0.036, respectively). Besides, PD-1 mRNA levels were not altered by nivolumab (**Figure 2D**, P= 0.408). These findings suggest that nivolumab blocks PD-1 on the surface of CIK cells. Interestingly, nivolumab caused a significant increase in the levels of CD4^+^ CD25^+^ CIK cells expressing Foxp3 (**Figure 2E**, P= 0.044).

### Nivolumab Affected the Cell Viability of Crizotinib-Treated NCI-H2228

**Figure 3:**
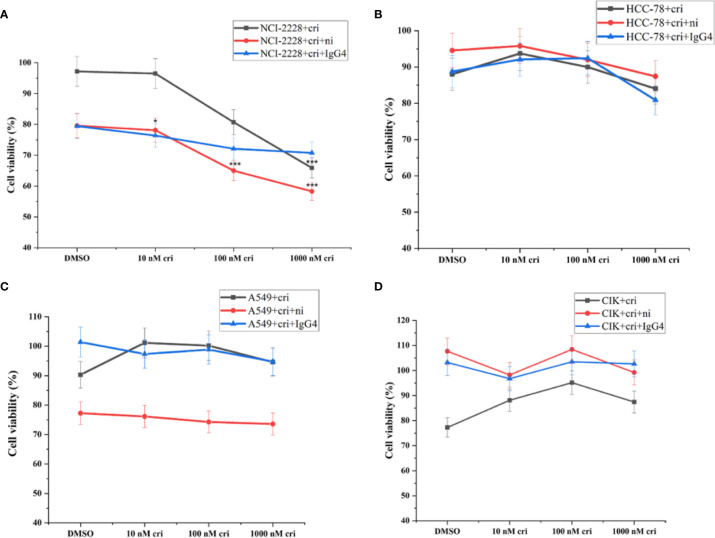
The tumor cell viability after the combination of nivolumab and crizotinib in the absence of CIK cells and CIK cells viability after the combination of nivolumab and crizotinib in the absence of tumor cells. **(A–C)** Tumor cells were pretreated with 20 ug/mL nivolumab for 24 h prior to incubation with crizotinib for 24 h. **(D)** CIK cells were pretreated with 20 ug/mL nivolumab for 24 h prior to incubation with crizotinib for 24 h. Each experiment repeated 3 times and CIK cells derived from four donors. All data are shown as the mean ± SD. *representative of three independent experiments. *P < 0.05, ***P < 0.001 vs. NCI-H2228 treatedwith DMSO control. One‐way ANOVA followed by the Tukey–Kramer *post hoc* test was performed. CIK cells derived from three healthy donors.

Considering that nivolumab may reduce the tumor cell viability, we performed the CCK-8 assay and found that the combined treatment of nivolumab with crizotinib significantly impaired the viability of NCI-H2228 cells (**Figure 3A**). Primarily, a significant change was observed when 20 μg/mL nivolumab and 100 nM crizotinib/1000 nM crizotinib were administered in NCI-H2228 cells compared to DMSO control (**Figure 3A**, 65.0% ± 3.9% vs. 97.2% ± 3.0%, p < 0.001; 58.3% ± 3.1% vs. 97.2% ± 3.0%, p < 0.001, respectively). However, no such change was observed in the HCC-78 and A549 cell lines as well as CIK cells, suggesting that this effect is cell line (or EML4-ALK variant 3) specific (**Figures 3B–D**).

### Nivolumab and Crizotinib Displayed an Additive Effect on the Cytotoxicity

**Figure 4:**
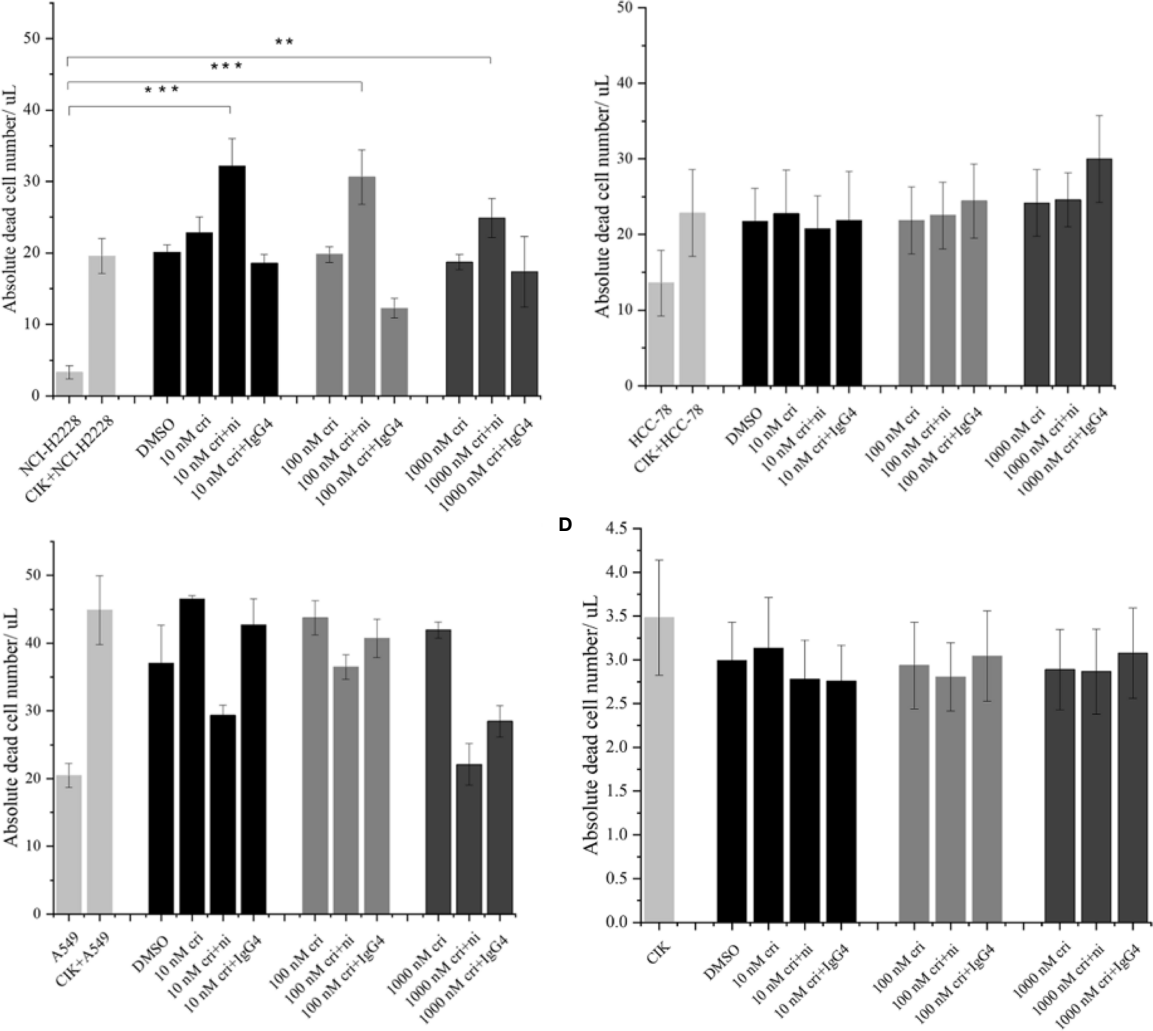
The absolute dead cell concentration after the combination of nivolumab and crizotinib. **(A–C)** The absolute number of dead cells in NSCLC cell lines after the co-culture with 24 h nivolumab-pretreated CIK, and in the presence of crizotinib (for 24 h) were detected by flow cytometry. NSCLC cells were labelled with CFSE and stained with dead cell Dye Hoechst 33258 before data was recorded in the flow cytometer. Dead CFSE^+^ tumor cells were analyzed by count beads and recorded by absolute cells/μL. **(D)** Similarly, 24 h nivolumab-pretreated CIK incubated with crizotinib (for 24 h) in the absence of tumor cells were stained with dead cell Dye Hoechst 33258 and detected by flow cytometry. Each experiment repeated 3 times and CIK cells derived from three donors. Data from three independent experiments were shown as mean ± SD. Statistical analysis was performed using one‐way ANOVA followed by the Tukey–Kramer *post hoc* test. **p < 0.01, ***p < 0.001 vs. untreated NCI-H2228.

To assess the absolute quantification of dead tumor cells (that may directly reflect the cytotoxic activity of CIK cells), we pre-labelled the NSCLC cells with carboxyfluorescein diacetate succinimidyl ester (CFSE) and co-cultured with nivolumab-pretreated CIK cells at an effector-target ratio of 10:1 in the presence of crizotinib. The absolute number of dead NCI-H2228 cells was found to be significantly elevated after combining 10 nM crizotinib/100 nM crizotinb/1000 nM crizotinb with nivolumab compared to the NCI-H2228 control (32.2 ± 3. 9 cells/μL vs. 3.3 ± 0.9 cells/μL, P< 0.001; 30.6 ± 3.8 cells/μL vs. 3.3 ± 0.9 cells/μL, P< 0.001; 24.9 ± 2.7 cells/μL vs. 3.3 ± 0.9 cells/μL, P = 0.004, respectively) (**Figure 4A**). Here again, two other cell lines (HCC-78 and A549) showed weaker sensitivity to CIK cytotoxicity when combined with crizotinib and nivolumab (**Figures 4B, C**). Meanwhile, this combination strategy did not influence the dead cell number in CIK cells (**Figure 4D**).

### Cumulative Effect of PD-1 Inhibitor With Crizotinib Influenced Interferon-γ and Intracellular Granzyme B

**Figure 5:**
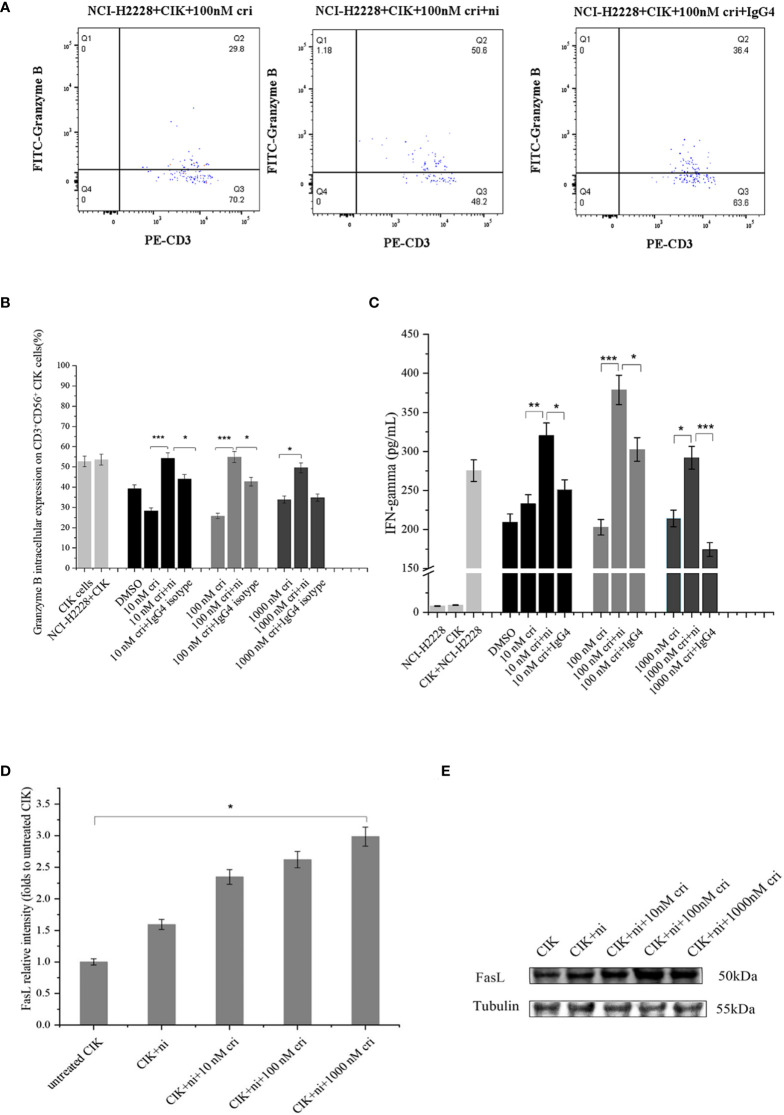
Effects of a combination of blocking PD-1 immune checkpoint and crizotinib on CIK-derived IFN-γ, granzyme B in CD3^+^ CD56^+^ CIK cells. CIK cells were pretreated in the presence or absence of 20 ug/ml nivolumab. At 24 h post mAb treatment in CIK cells, CIK cells were co-cultured with NCI-H2228.**(A)** Intracellular granzyme B expression in CD3^+^ CD56^+^ CIK cells in the presence of PD-1 blockade against NCI-H2228 cells is shown in a dot plot, measured by flow cytometry at different levels of crizotinib. **(B)** Bar plots depict the percentage of intracellular granzyme B expression on CIK cells and **(C)** IFN-γ levels from CIK cells after treatment with a combination of PD-1 blockade and crizotinib on NCI-H2228 target cells. *p < 0.05, **p < 0.01, ***p < 0.001 vs. crizotinib monotherapy or IgG4 isotype control. **(D)** FasL expression was observed by Western blots. CIK cells were treated with nivolumab for 24 h prior to the addition of different concentrations of crizotinib. **(E)** The relative intensities of FasL expression normalized to each Tubulin control without treatment were defined as 1.0. Then, the other relative intensity (folds) was presented. Data were presented as means ± SD of the results from three independent experiments. *p < 0.05 vs. CIK control without any treatment. Each experiment repeated 3 times and CIK cells derived from three donors and the data were shown as mean ± SD. Statistical analysis was performed using one‐way ANOVA followed by the Tukey–Kramer *post hoc* test.

To investigate the potential variations in immune markers and cytokines primarily associated with the cytotoxicity of CIK cells, we subsequently examined IFN-γ expression (by ELISA) and intracellular expression of granzyme B by flow cytometry (**Figures 5A, B**) and IFN-γ expression by ELISA (**Figure 5C**). CIK cells responded to a combination of PD-1 blockade and crizotinib against NCI-H2228 with a strong secretion of IFN-γ and a significant increase in granzyme B in the CD3^+^ CD56^+^ subpopulation of CIK cells. Specifically, in combination with CIK cells, anti-PD-1 and 10 nM crizotinib showed a significant increase compared to either crizotinib or isotype control and/or crizotinib alone (320.3 ± 48.2 vs. 232.8 ± 41.6 pg/mL, P= 0.004; 320.3 ± 48.2 vs. 251.1 ± 40.1 pg/mL, P= 0.022). However, no significant change was observed in the HCC-78 and A549 cell lines (**Supplementary Figure 1**).

### Nivolumab Combined Crizotinib Upregulated the Intrinsic FasL Expression in CIK Cells

Considering that cell surface FasL expression is specific to the immune system, we investigated FasL surface expression on the CIK cells. The analysis showed a low level of surface FasL (less than 5%), which was further verified using an additional methodological approach (Western blot) (**Figures 5D, E**). Importantly, nivolumab combined with 1000 nM crizotinib resulted in significant upregulation of intrinsic FasL expression compared to the untreated CIK control (the band intensity 3.0 ± 0.7 vs. 1.0 ± 0.0, P= 0.028).

### CIK Cells Ameliorated the Hepatoxicity in CCL-13 Cell Line Concurrent Incubation With Nivolumab Combined With Crizotinib

To assess the hepatoxicity, LDH release assay was performed. After incubation with CIK cells, cytotoxicity of the simultaneous combination of 1000 nM crizotinib and nivolumab was significantly reduced compared to cytotoxicity without CIK (**Supplementary Figure 2A**, P<0.001). Furthermore, the combination of CIK cells with crizotinib and nivolumab significantly decreased the mRNA expression of C - reactive protein (CRP), which is known to be associated with reactive oxygen species (ROS) (**Supplementary Figure 2B**, P=0.047).

## Discussion

Given cancer is quite a complex disease and monotherapies pose a serious challenge regarding patient survival rate, the development of new combination therapies has partially improved patient outcomes. The combined use of therapeutic agents within combination therapy has proven to be advantageous (over monotherapy) in several ways, such as enhanced efficacy, reduced side effects, and minimal drug resistance. Of clinical importance, several cancers have been reported to optimize this therapeutic approach, such as renal cell carcinoma, melanoma, Hodgkin’s lymphoma, and breast cancer to name a few (31–34). In particular, non-small cell lung cancer (NSCLC) is one of the prominent cancers where a number of studies documenting the clinical outcomes of mono and/or combination therapies have been reported (www.cancer.gov). However, there are serious concerns when the combination of compounds showed a synergistic effect in the preclinical models while failing in the clinical application. As mentioned above, the clinical trial with the combination of crizotinib (ALK inhibitor) and nivolumab (PD-1 inhibitor) was terminated due to safety concerns in NSCLC, while the same combination of compounds showed an improved antitumor response with favorable side-effect tolerability in the *in vivo* model.

In this current study, we investigated whether the inclusion of alternative treatments (for instance, cytokine-induced killer cells) and variable genetic backgrounds (NCI-H2228: EML4-ALK, A549: KRAS mutation, HCC-78: ROS1 rearrangement) within the same cancer type can help to provide an advantageous therapeutic paradigm in NSCLC. In the context of genetic variations, mutations in a few genes (KRAS, EGFR, ALK, ROS1, BRAF, RET, MET, NTRK) have been shown to be clinically relevant and proven to be pivotal to identify patient subgroups with early/advanced-stage patients. Moreover, accumulating data from different ethnic groups has confirmed these previous findings on the NSCLC-associated mutational paradigm and highlighted the potential genetic differences across different racial groups (35–37), given that CIK cells have previously shown positive outcome in NSCLC treatment, and nivolumab has been shown to be highly promising in NSCLC (38). As a proof of concept, we first confirmed that all cell lines expressed PD-L1, but at different levels. Next, we demonstrated that a combination of nivolumab and crizotinib improved the immune function of CIK cells compared to monotherapy with crizotinib, especially in the NSCLC-specific cell line NCI-H2228. We further first verified that PD-1 surface expression on CIK cells decreased dramatically after the treatment with nivolumab and it had no effect on PD-1 mRNA expression in CIK cells. Our findings revealed that PD-1 inhibitors only conjugate on the surface of CIK cells without PD-1 mRNA alternation. Nevertheless, nivolumab was found to bind to T lymphocytes for more than 20 weeks in NSCLC patients, even after stopping treatment, suggesting a sustained therapeutic effect (39). Although one clinical trial reported that treatment of NSCLC patients with anti-PD-1 antibodies nivolumab expanded the levels of effector T cells expressing the costimulatory molecules CD28, CD27, and ICOS (40), we did not observe the similar immuno-logical events (**Figure 2B**). Importantly, our study showed that nivolumab caused an increase in the proportion of Foxp3^+^ CD4^+^ CD25^+^ CIK cells (**Figure 2E**), which is consistent with the previous reports (41). This suggests compensatory expression of immune molecules after PD-1 blockade. Second, we observed that the blocking of the PD-1/PD-L1 pathway by nivolumab increased the cytotoxicity of CIK cells targeted to NCI-H2228 rather than HCC-78 and A549 cells. Additionally, the evidence obtained from multiple markers in our study, such as increased production of IFN-γ from CIK cells, activation of FASL in CIK cells, and increased intracellular expression of granzyme B on CD3^+^ CD56^+^ NKT CIK cells, suggest that the combination of nivolumab and crizotinib accelerates the release of immune-related effector molecules. Previously, CIK cells were reported to express a high percentage of granzyme B, a serine protease that together with another protein perforin mediates apoptosis in target cells (42). Similarly, an addition of anti-PD-1 inhibitor has been shown to enhance the granzyme B expression in DC-CIK cells (43). Considering this, we assessed and found an increased level of intracellular granzyme B in PD-1 activated CIK cells after being combined with an ALK inhibitor. FasL, expressed on activated T cells and natural killer (NK) cells, poses another way to kill cancer cells by recruiting Fas-associated protein with DD (FADD) and activating caspase pathways. Notably, Verneris et al. found that CIK cells are resistant to apoptosis by expressing anti-apoptotic genes and can synthesize FasL to induce Fas-dependent apoptosis of sensitive tumor cells (44). In our current study, we are reporting for the first time that a combination of dose-dependent crizotinib and nivolumab can also promote FASL expression in CIK cells, overall suggesting that CIK cells use a different apoptosis pathway to eliminate NSCLC cells *in vitro*. Thus, our findings about the elevated granzyme B levels (along with activation of FASL) in a similar experimental setup suggest a yet unknown mechanism that may be of interest for future NSCLC immunotherapy.

Since our analysis showed predominance in NCI-H2228 compared to HCC-78 and A549 cells, we can assume three possibilities: (1) heterogeneity across cancer cell lines may lead to major discrepancies in the experimental data, as previously discussed by Sharma et al. (45), (2) origin of cell lines, A549 is a lung adenocarcinoma cell line with a mesenchymal signature and is known to be more resistant to the drugs targeting PI3K‐AKT pathway (46), and (3) the presence of intrinsic PD-1 protein expression in NSCLC cells that may inhibit tumor cell proliferation and might exert resistance to PD-1/PD-L1 blockades (47). Nevertheless, our study supports the idea that combination therapies have enormous potential if they are optimized on a patient-by-patient basis. Like other cancers, the clonal heterogeneity and the existence of cancer stem cells (CSCs) in lung cancer also continue to be subjects of discussion (48). In an interesting study, six small cell lung cancer (SCLC) cell lines were used to investigate whether CD133 or CD87 could be a potential marker for CSCs (49). The authors showed that both CD133 and CD87 are expressed in the SBC-7 cell line as inadequate markers of CSCs and might be beneficial for predicting chemotherapy resistance. Recently, a study confirmed the identification of CSCs and showed that PD-L1^+^ CSCs are strongly associated with altered T cell phenotypes and, in particular, the frequency of regulatory molecule-expressing T cells in metastatic lymph nodes of NSCLC patients (50). Of note, our current study mainly concerns enhancing the cytotoxicity and did not emphasize CSCs; however, we cannot exclude the contribution of lung cancer associated CSCs. Certainly, future studies utilizing CIK/ALK/PD inhibitors axis in context with the tumor microenvironment of NSCLCs will be of interest. Here, it is also important to mention CheckMate 370, which was discontinued due to severe hepatotoxicity in 2018. In this context, we also performed analysis to determine whether CIK cells can alleviate the hepatoxicity after incubation with ALK inhibitor and PD-1 inhibitor simultaneously. To achieve this, we used the hepatocyte-like cell line CCL-13, which exhibits the liver function of primary human hepatocytes, and found that: (1) after incubation with CIK cells, the cytotoxicity of the simultaneous combination of crizotinib and nivolumab was significantly reduced as detected by LDH assay. (2) The combination of CIK cells with crizotinib and nivolumab significantly decreased the mRNA expression of C-reactive protein (CRP), which is known to associate with reactive oxygen species (ROS). Therefore, it is reasonable to conclude that CIK cells may have mitigated with hepatotoxicity. In broader terms, the limitations of our study are also worth mentioning, such as (A) we did not investigate the combination of DC-CIK and ALK inhibitor with PD-1 inhibitor, which may exert more potent cytotoxic effects than CIK cells as shown in previous studies (43). (B) The data from a few additional cell lines (e.g., with KRAS, EGFR, and p53 mutation status) would be valuable in this analysis. (C) Our findings require *in vivo* validation. Despite these facts, our data as preclinical model provide an insight about the antitumor immune response by CIK cells in combination with crizotinib and nivolumab.

Based on our results and the existing literature, a few perspectives can be considered for NSCLC, for example, (a) given that response to therapies and improved survival varies significantly in NSCLC subtype, a clear emphasis on precision medicine/therapy should be considered. (b) With the development of second- and third-generation ALK inhibitors, their synergistic effects in preclinical studies, mainly on experimental models associated with NSCLC mutations, may improve their efficacy and resistance problem. (c) As recently proposed, the combinatorial aspect of CIK cell therapy combined with other contemporary anti-cancer therapies in a complementary (rather than competitive) manner may be the key to combat cancer (51), also applies in NSCLC. (d) Identifying a subpopulation of NSCLC patients (with ALK rearrangement) who may benefit from the combination of an ALK inhibitor and PD-1 inhibitor must be stratified according to known risk factors (52) and potential biomarkers (e.g., C-reactive protein levels) (53). In a broader prospective, though different cancers are represented with their unique mutational/epigenomic profiles, they still share few common/overlapping signaling pathways (54) and, therefore, it is important to stratify the uniqueness of each cancer type already in the preclinical models.

## Conclusions

CIK cells in combination with crizotinib and nivolumab can enhance the antitumor immune response through FasL activation, leading to increased IFN-γ and granzyme B, but only in NCI-H2228 cells with EML4-ALK rearrangement. Hence, our study suggests that genetic background plays a significant role, and the combination therapies should be optimized by considering underlying factors (genetic background and immune response) on a patient-by-patient basis.

## Data Availability Statement

The raw data supporting the conclusions of this article will be made available by the authors, without undue reservation.

## Ethics Statement

The studies involving human participants were reviewed and approved by Ethics Committee of the University of Bonn. The patients/participants provided their written informed consent to participate in this study.

## Author Contributions

Conceptualization, YL and IS-W; methodology, YL and XW; validation, YL, XW, AS; formal analysis, YL; data curation, YL, AS, and IS-W; writing—original draft preparation, YL, AS, and IS-W; writing—review and editing, all co-authors; supervision, IS-W, HW, DS, and ME; project administration, IS-W. All authors contributed to the article and approved the submitted version.

## Conflict of Interest

The authors declare that the research was conducted in the absence of any commercial or financial relationships that could be construed as a potential conflict of interest.

## Publisher’s Note

All claims expressed in this article are solely those of the authors and do not necessarily represent those of their affiliated organizations, or those of the publisher, the editors and the reviewers. Any product that may be evaluated in this article, or claim that may be made by its manufacturer, is not guaranteed or endorsed by the publisher.
