# Supplementary material for: A Combination of Cytokine-Induced Killer Cells With PD-1 Blockade and ALK Inhibitor Showed Substantial Intrinsic Variability Across Non-Small Cell Lung Cancer Cell Lines
Source: Front Oncol. 2022 May 11;12:713476. doi: 10.3389/fonc.2022.713476 (PMC9130779; doi:10.3389/fonc.2022.713476)
Supplement: Supplementary file 1 [file DataSheet_1.docx]

Supplementary Material


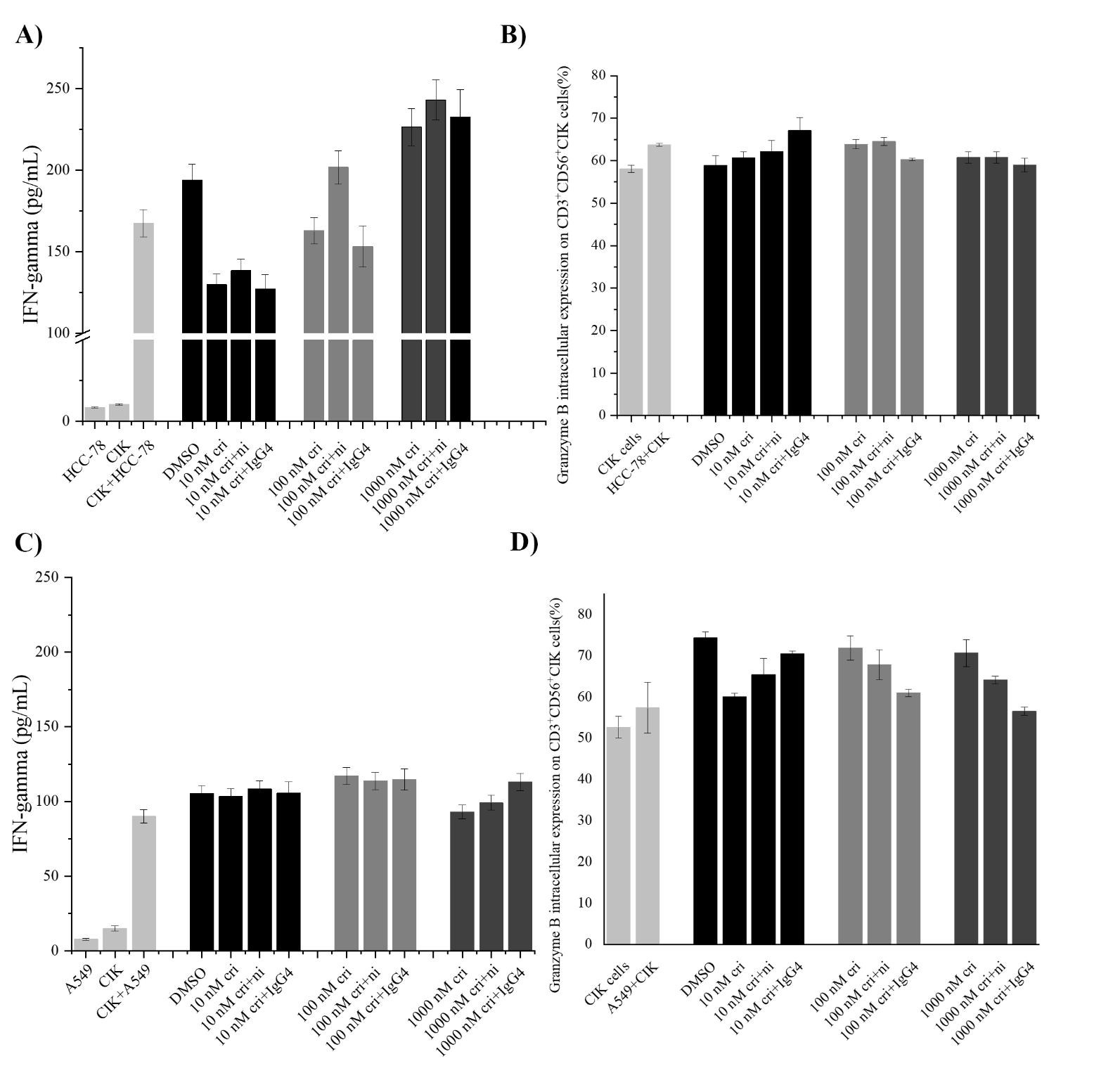


**Supplementary Figure 1.** Effects of a combination of blocking PD-1 immune checkpoint and crizotinib on CIK-derived IFN-γ, granzyme B in CD3+ CD56+ CIK cells. a) The level of IFN-γ from CIK cells co-culturing with HCC-78 cells, b) granzyme B expression in CIK cells co-culturing with C-78 cells, c) the level of IFN- γ from CIK cells co-culturing with A549 cells, d) granzyme B expression in CIK cells co-culturing with A549 cells.


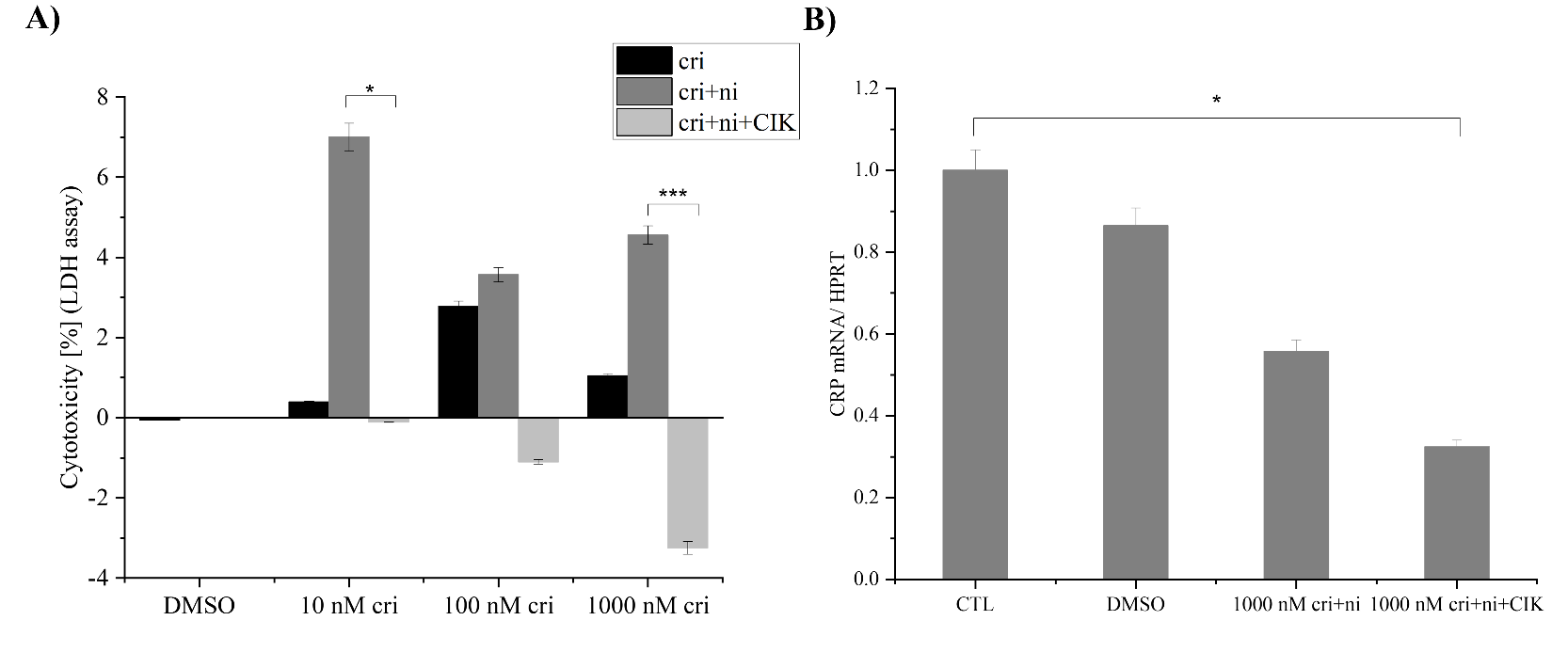


**Supplementary Figure 2.** The hepatoxicity of a combination of blocking PD-1 immune checkpoint and crizotinib on hepatocyte-like cell line CCL-13 in the presence of CIK cells simultaneously. a) The percentage of the cytotoxicity detected by lactate dehydrogenase (LDH) release for 16 hours co-culture with CIK cells and CCL-13 in the presence of crizotinib and nivolumab simultaneously, b) the mRNA expression of C-Reactive Protein (CRP) in CCL-13 co-culture with CIK cells in the presence of crizotinib and nivolumab simultaneously for 24 hours. Each experiment was repeated 3 times, CIK cells were derived from three donors and the E:T ratio is 1:1. The data are shown as mean ± SD. Statistical analysis was performed using one‐way ANOVA followed by the Tukey–Kramer post hoc test. * p < 0.05 , *** p < 0.001.
